# Mecanismos del COVID-19 en el cuerpo humano: Lo que sabemos hasta ahora

**DOI:** 10.1159/000521507

**Published:** 2022-01-24

**Authors:** Ashutosh Kumar, Ravi K. Narayan, Pranav Prasoon, Chiman Kumari, Gurjot Kaur, Santosh Kumar, Maheswari Kulandhasamy, Kishore Sesham, Vikas Pareek, Muneeb A. Faiq, Sada N. Pandey, Himanshu N. Singh, Kamla Kant, Prakash S. Shekhawat, Khursheed Raza, Sujeet Kumar

**Affiliations:** ^a^Red de Investigación de Trastornos Etiológicamente Elusivos (EEDRN), Nueva Delhi, India; ^b^Departamento de Anatomía, (AIIMS), Patna, India; ^c^Departamento de Anatomía, Instituto de Ciencias Médicas de las Islas Andamán y Nicobar, Port Blair, India; ^d^Centro de Investigación del Dolor de Pittsburgh, Facultad de Medicina, Universidad de Pittsburgh, Pittsburgh, Pennsylvania, Estados Unidos; ^e^Departamento de Anatomía, Instituto de Posgrado de Educación e Investigación Médica (PGIMER), Chandigarh, India; ^f^Escuela de Ciencias Farmacéuticas, Universidad Shoolini, Solan, India; ^g^Departamento de Anestesiología y Medicina Crítica, Escuela de Medicina, Universidad Johns Hopkins, Baltimore, Maryland, Estados Unidos; ^h^Departamento de Bioquímica, Colegio Médico Maulana Azad (MAMC), Nueva Delhi, India; ^i^Departamento de Anatomía, Instituto de Ciencias Médicas de la India (AIIMS), Mangalagiri, Vijayawada, India; ^j^Centro de Ciencias Cognitivas y del Cerebro, Instituto Indio de Tecnología Gandhinagar, Gandhinagar, Gujarat, India; ^k^Universidad de Nueva York (NYU) Langone Health Center, Escuela de Medicina Robert I. Grossman de la NYU, Nueva York, New York, Estados Unidos; ^l^Departamento de Zoología, Universidad Hindú de Banaras (BHU), Varanasi, India; ^m^Departamento de Biología de Sistemas, Centro Médico Irving de la Universidad de Columbia, Nueva York, New York, Estados Unidos; ^n^Departamento de Microbiología, Instituto de Ciencias Médicas de la India (AIIMS), Bathinda, India; ^o^Departamento de Hematología Clínica, Instituto Nacional de Ciencias Médicas, Jaipur, India; ^p^Departamento de Anatomía, Instituto de Ciencias Médicas de la India (AIIMS), Deoghar, India; ^q^Centro de Proteómica y Desarrollo de Fármacos, Instituto de Biotecnología Amity, Universidad Amity, Maharashtra, India

## Abstract

Ha transcurrido más de un año y medio desde el inicio de la pandemia debida a la enfermedad por coronavirus 2019 (COVID-19), y el mundo se esfuerza por contenerla. Al ser causada por un virus hasta ahora desconocido, en el periodo inicial hubo una escasez extrema de conocimientos sobre los mecanismos de la enfermedad, lo que dificultó las medidas preventivas y terapéuticas contra el COVID-19. En un esfuerzo por comprender sus mecanismos patogénicos, se han llevado a cabo extensos estudios experimentales en todo el mundo con experimentos basados en cultivos celulares, organoides de tejidos humanos y modelos animales, enfocados en varios aspectos de la enfermedad, como las propiedades del virus, su tropismo tisular y su patogénesis específica en ciertos órganos, la implicación de los sistemas fisiológicos y la respuesta inmunitaria humana contra la infección. El enorme conocimiento científico acumulado sobre todos los aspectos del COVID-19 cambió el escenario, de una gran desolación, a la esperanza. A pesar de que se han producido avances espectaculares en todos estos aspectos, quedan múltiples lagunas de conocimiento que deben abordarse en futuros estudios. Además, desde el inicio de la primera oleada de COVID-19 han surgido en todo el mundo múltiples variantes del coronavirus tipo 2 del síndrome respiratorio agudo severo (SARS-CoV-2), que parecen tener mayor transmisibilidad/virulencia y capacidad de escape inmunológico que la cepa de tipo salvaje. En esta revisión narramos los avances realizados desde el inicio de la pandemia en nuestros conocimientos sobre los mecanismos del COVID-19 en el cuerpo humano, incluyendo las interacciones virus-huésped, las manifestaciones pulmonares y sistémicas, las desregulaciones inmunológicas, las complicaciones, la vulnerabilidad específica del huésped y las consecuencias a largo plazo en los supervivientes. Además, ofrecemos una breve revisión de las pruebas actuales que explican los mecanismos moleculares que confieren mayor transmisibilidad, virulencia y capacidad de escape inmunológico a las variantes emergentes del SARS-CoV-2.

## Introducción

La actual pandemia de la enfermedad por coronavirus 2019 (COVID-19) ha cobrado un gran número de vidas humanas en todo el mundo (∼4.8 millones hasta el 8 de octubre de 2021, según datos de la OMS). El COVID-19 es causado por el coronavirus tipo 2 del síndrome respiratorio agudo severo (SARS-CoV-2) [1], un virus envuelto, con ARN monocatenario de sentido positivo, que pertenece al género de los betacoronavirus (BCoV). Existen otros BCoVs, como el SARS-CoV-1 y el coronavirus del síndrome respiratorio de Oriente Medio (MERS-CoV), que han causado las epidemias de síndrome respiratorio SARS-2002/2003 y MERS-2012, respectivamente [1].

La extrema escasez de conocimientos, especialmente en el periodo inicial, sobre la interacción de la nueva cepa de coronavirus, el SARS-CoV-2, con el huésped dificultó enormemente el manejo preventivo y terapéutico de la pandemia. Sin embargo, la gigantesca investigación realizada en todo el mundo ha generado evidencias científicas rigurosas sobre todos los aspectos del COVID-19 a una velocidad y una escala sin precedentes. Los hechos emergentes sobre los mecanismos patogénicos del COVID-19 han ayudado a frenar la propagación incontrolada de la pandemia mediante el desarrollo de varias medidas preventivas, incluyendo vacunas efectivas, y un mejor manejo terapéutico. Aunque aún no existe ningún fármaco eficaz para el COVID-19, el estado actual de la investigación hace concebir esperanzas sobre el futuro. La aparición de múltiples variantes de SARS-CoV-2 con mayor transmisibilidad/virulencia y capacidad de escape inmunológico es un giro desafortunado en el curso actual (2020–2021) de la pandemia. Surgen evidencias que pueden explicar una mejor interacción entre el virus y el huésped y la mayor capacidad de escape inmunitario de las variantes [2, 3, 4]; sin embargo, aún se desconoce si las variantes han alterado su patogénesis para un tipo de tejido u órgano específico. En esta revisión, analizamos con precisión evidencias recientes sobre los mecanismos patogénicos del COVID-19 en humanos, incluyendo las interacciones virus-huésped, manifestaciones pulmonares y sistémicas, desregulaciones inmunológicas, complicaciones, la vulnerabilidad específica del huésped y los problemas de salud a largo plazo en los supervivientes. El artículo tiene como objetivo ofrecer una comprensión completa de los mecanismos patogénicos clave que conducen a las manifestaciones clínicas y a la evolución de los pacientes en el COVID-19, y destacar las lagunas en los conocimientos que pueden requerir mayor atención de los investigadores. Además, se analizan brevemente las evidencias actuales sobre los mecanismos moleculares que confieren mayor transmisibilidad, virulencia y capacidad de escape inmunológico a las variantes emergentes del SARS-CoV-2.

## Sintomatología del COVID-19

**Fig. 1:**
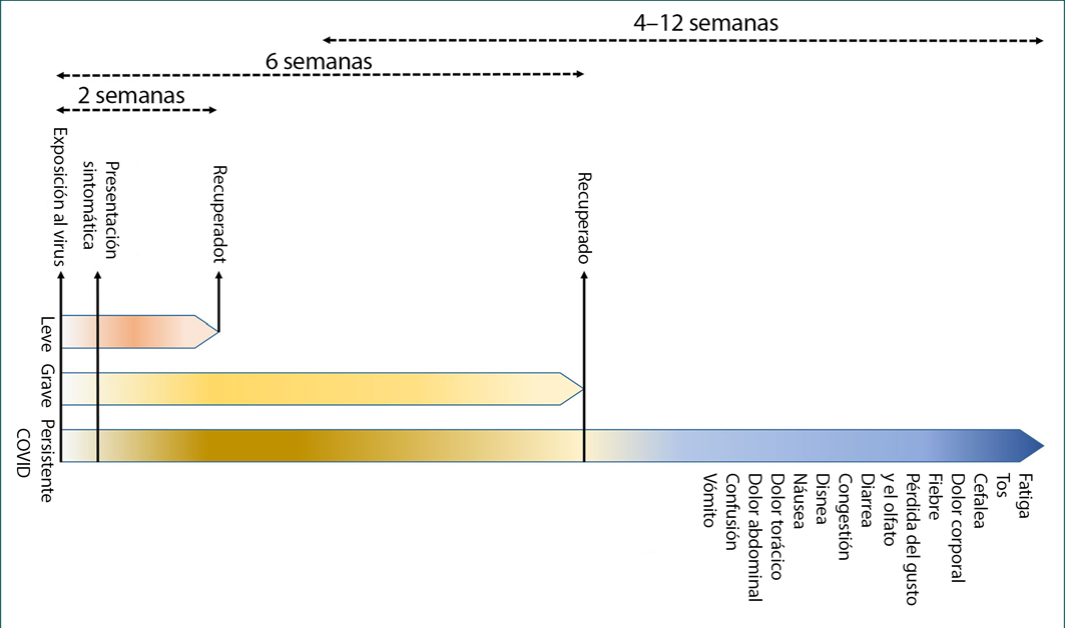
Sintomatología de la enfermedad por coronavirus 2019 (COVID-19). El inicio de los síntomas clínicos se produce en promedio 5–6 días después de la exposición, y normalmente, los enfermos con síntomas leves se recuperan en dos semanas; sin embargo, en casos graves, la recuperación puede extenderse hasta seis semanas. En algunos pacientes la enfermedad puede persistir o, tras la recuperación completa, pueden aparecer otras molestias, lo que se conoce como «COVID persistente». El «COVID persistente» se caracteriza principalmente por la presencia de fatiga, dolor de cabeza, disnea y anosmia, que pueden persistir durante 4–12 semanas.

El COVID-19 se ha descrito principalmente como una infección causante del síndrome respiratorio agudo severo (SARS); sin embargo, son muy habituales las manifestaciones sistémicas que afectan a otros órganos, incluido el sistema nervioso central (SNC) [5] (Tabla 1). El inicio de los síntomas se produce en promedio 5–6 días después de la exposición; normalmente, quienes tienen síntomas leves se recuperan en dos semanas; sin embargo, en los casos graves, la recuperación puede extenderse hasta seis semanas (Figura 1). Cabe destacar que en algunos pacientes, independientemente de la gravedad de la enfermedad, los síntomas pueden persistir o reaparecer durante semanas o meses tras la recuperación inicial [17]. En muchos supervivientes se ha reportado la persistencia de la enfermedad o la aparición de nuevas dolencias tras la recuperación completa, lo que se conoce como «COVID persistente» [17, 18, 19] (Figura 1). La presentación de datos clínicos en los pacientes con COVID-19 ha revelado algunos hechos interesantes; del total de casos, aproximadamente 80% son asintomáticos o presentan síntomas leves, mientras que ∼14% desarrollan síntomas graves, como neumonía, ∼5% desarrollan síntomas críticos, como shock séptico, insuficiencia respiratoria o falla multiorgánica, y ∼2% de los pacientes mueren a causa de la enfermedad [20]. La mortalidad es comparativamente mucho mayor en las personas de edad avanzada y con comorbilidades [21, 22].

De acuerdo con la revisión de la literatura actual, el «COVID persistente» se caracteriza por síntomas de fatiga, dolor de cabeza, disnea y anosmia. Es más probable que se produzca ancianos, personas con alto índice de masa corporal y de sexo femenino, y los síntomas pueden persistir durante 4–12 semanas. Además, los pacientes con COVID que experimentaron más de cinco síntomas durante la primera semana de la enfermedad podrían tener una probabilidad significativamente mayor de desarrollar «COVID persistente» (odds ratio = 3.53 (2.76–4.50)) [17]. Un estudio reciente indica que algunos síntomas del «COVID persistente» pueden prolongarse más allá de un año, en particular la fatiga, la disnea y síntomas neurológicos como la ansiedad y la depresión [23].

## Interacciones entre el virus y el huésped

### Entrada del SARS-CoV-2 en las células del huésped

**Fig. 2:**
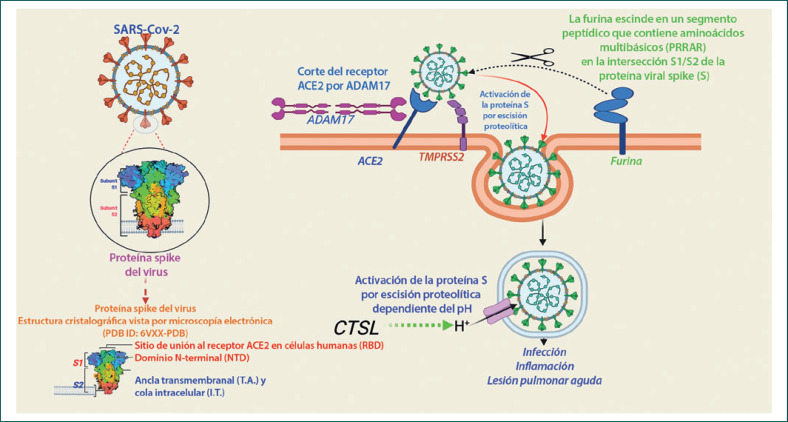
Esquema de la entrada del coronavirus tipo 2 del síndrome respiratorio agudo severo (SARS-CoV-2) en las células humanas. La entrada del SARS-CoV-2 en las células humanas es mediada por un receptor de superficie celular, la enzima convertidora de angiotensina-2 (ACE2). La ACE2 se une al dominio de unión al receptor (RBD) de la proteína spike (S) del SARS-CoV-2. Además, para que el virus entre en la célula huésped, la imprimación de la proteína «S» para su fusión con la membrana de la célula huésped se realiza mediante proteasas de la célula huésped, lo que implica la escisión de la proteína «S» por serina proteasas, la serina proteasa transmembrana 2 (TMPRSS2) o la catepsina B o L (CTS-B o -L), y la furina presente en la membrana de la célula huésped. La CTS-B o -L actúa principalmente en el interior de los endosomas. El sitio de corte de la furina (PRRAR), presente en la intersección de S1 y S2, se considera una adquisición evolutiva en el SARS-CoV-2.

La entrada del SARS-CoV-2 en las células humanas es mediada por un receptor de superficie celular, la enzima convertidora de angiotensina-2 (ACE2) [24] (Figura 2). La ACE2 se une al dominio de unión al receptor (RBD) de la proteína spike (S) del SARS-CoV-2. Además, para que el virus pueda entrar en la célula huésped, la imprimación de la proteína SSC se considera esencial para su fusión con la membrana de la célula huésped, lo que implica la escisión de la proteína «S» por serina proteasas como la llamada serina proteasa transmembrana 2 (TMPRSS2) o por la catepsina B o L (CTS-B o -L) y la furina presentes en la membrana de la célula huésped [1] (Figura 2) (revisado en [1]).

Se sabe que ACE2 es un receptor de entrada a la célula huésped para algunos otros CoV, como el SARS-CoV-1 y el HCoV-NL-63. También se sabe que TMPRSS2 y CTS-L actúan como vía de entrada para SARS-CoV-1 y otros virus relacionados con el SARS [1]. Sin embargo, la inclusión de la furina parece ser una adquisición evolutiva en el SARS-CoV-2 en comparación con otros virus del SARS (revisado en [1]). El corte por la furina requiere la inserción de un segmento peptídico, el sitio de corte de furina (FCS), que contiene aminoácidos multibásicos (PRRAR) en la intersección S1/S2 de la proteína «S», que no están presentes en el SARS-CoV-1 ni en otros virus relacionados con el SARS [25]. En particular, se sabe que el FCS está presente en muchos otros CoV, como el MERS-CoV, el HKU1-CoV y el OC43-CoV [26]. Se especula que la inclusión del FCS en el genoma del SARS-CoV-2 contribuye a su alta infectividad y transmisibilidad en comparación con otros virus del SARS [25]. El FCS también se ha observado en virus de la influenza y se considera que contribuye a su virulencia [27]. En la actualidad, hay limitada evidencia de si la inclusión del FCS contribuye a la virulencia del SARS-CoV-2 (revisado en [1]). En un estudio reciente, se descubrió que un mutante del SARS-CoV-2 que carecía de FCS en la proteína «S» presentaba una menor replicación en las células Calu3 (una línea celular respiratoria humana) y una progresión atenuada de la enfermedad en un modelo de patogénesis del COVID-19 en hámster [28].

Además de la ACE2, estudios recientes han identificado evidencia concreta de un receptor de entrada de células huésped alternativo para el SARS-CoV-2: la neuropilina-1 (NRP1) [25, 26]. La NRP1 se expresa abundantemente en múltiples tipos de tejidos en todo el cuerpo, con una expresión muy alta en las células endoteliales y epiteliales, en particular en el epitelio respiratorio y olfativo. Cantuti-Castelvetri et al. [29] demostraron que el NRP1 potencia la infectividad del SARS-CoV-2. Además, Daly et al. [30] demostraron que el fragmento S1 de la proteína spike, escindido por la furina, puede unirse directamente a la NRP1 de la superficie celular, y que el bloqueo de esta interacción mediante un inhibidor de moléculas pequeñas o anticuerpos monoclonales reduce eficazmente la infección por el SARS-CoV-2.

### Tropismo tisular y organotropismo

**Fig. 3:**
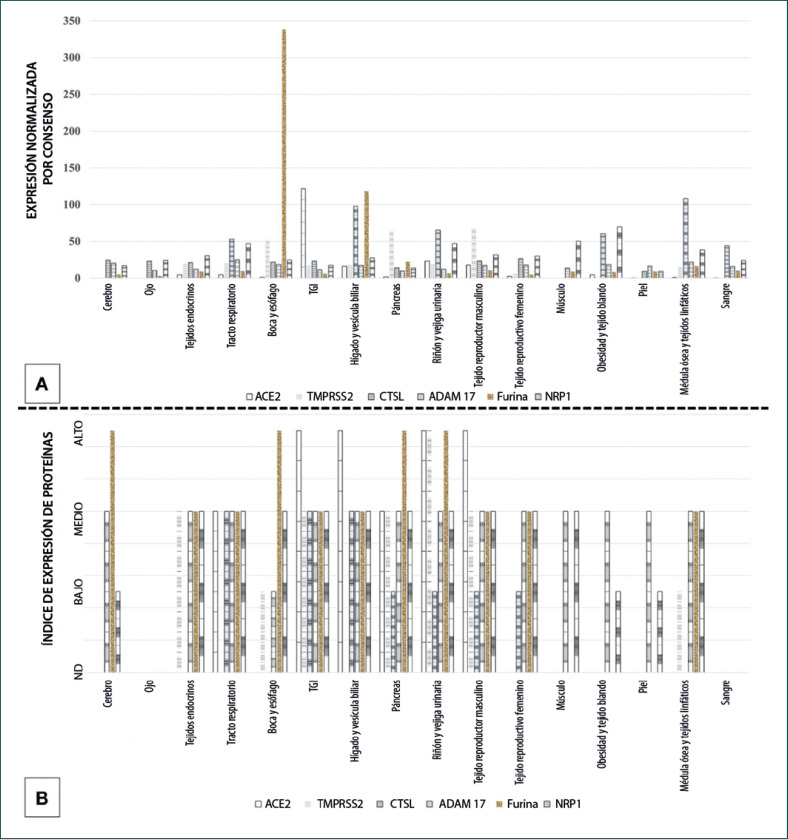
Expresión de los receptores de entrada a la célula huésped del coronavirus tipo 2 del síndrome respiratorio agudo severo (SARS-CoV-2) y de las proteasas asociadas con su entrada en varios tipos de tejidos humanos. (**a**) ARNm. (**b**) Proteína. Fuente de datos: Human Protein Atlas (https://www.proteinatlas.org/).

Los factores de entrada del SARS-CoV-2 en la célula huésped se expresan ampliamente en todos los tipos de tejidos en humanos [31, 32, 33]. Además, ACE2 se coexpresa junto con TMPRSS2/CTS-L en muchos tipos de tejidos, lo cual es esencial para la infección por el SARS-CoV-2 [32, 33]. El amplio tropismo tisular del SARS-CoV-2 se refleja en la diversidad de los síntomas observados en pacientes con COVID-19 [5]. El tropismo multiorgánico del SARS-CoV-2 también se ha confirmado en estudios con observaciones histopatológicas en muestras de tejido post mortem de pacientes infectados [34] y mediante la infección en laboratorio de organoides de tejido humano [35]. En la Figura 3 se describen los tipos de tejidos que pueden infectarse con SARS-CoV-2 en función de la expresión de los receptores de entrada de las células del huésped y de las proteasas del huésped asociadas con su ingreso.

### Secuestro de la maquinaria de la célula huésped

Estudios recientes han desentrañado los mecanismos moleculares impulsados por el SARS-CoV-2 para secuestrar la maquinaria de la célula huésped, en particular, para la síntesis de proteínas y los mecanismos de producción de energía [36, 37]. Los estudios in vitro han aportado evidencias sólidas de una extensa fosforilación de proteínas víricas del SARS-CoV-2 por parte del proteoma del huésped, implicada en la activación de las quinasas de la célula huésped y la señalización del receptor del factor de crecimiento (GFR), facilitando así el secuestro de la maquinaria proteica del huésped. La primera evidencia de este tipo publicada por Bouhaddou et al. [38] demostró que la infección por SARS-CoV-2 de las células del huésped promueve la activación de la caseína quinasa II (CK2) y de la proteína quinasa activada por mitógenos (MAPK) p38, así como la producción de diversas citocinas, lo que lleva a la desactivación de la quinasa dependiente de ciclinas (CDK) 1/2/5 y a la detención del ciclo celular [38]. Los autores también observaron una característica única del SARS-CoV-2 que era la menos conocida para los virus respiratorios: la infección viral indujo marcadamente la producción de protrusiones filopodiales distintivas que contenían CK2 y también partículas virales en desarrollo. Las protuberancias filopodiales parecían facilitar la transferencia del virus infeccioso a otras células del huésped. Este patrón de activación de la vía molecular por parte del SARS-CoV-2 podría explicar potencialmente los rasgos distintivos de las lesiones en los tejidos del huésped durante el COVID-19 grave, como la inflamación aguda, el daño a las células epiteliales y la disfunción endotelial vascular. Otra evidencia destacada se debe a Klann et al. [37], quienes demostraron que la infección por SARS-CoV-2 de una línea celular de epitelio colónico humano − células Caco-2 − activaba la señalización del GFR y sus vías descendentes. Estos autores también demostraron que una inhibición de la señalización del GFR impedía la replicación del SARS-CoV-2 en las células huésped [37]. Asimismo, otro estudio reciente con un modelo murino de COVID-19 ha sugerido que la toxicidad sistémica inducida por el SARS-CoV-2 provoca una regulación a la baja en la expresión de los genes que afectan los mecanismos de producción de energía en las células, como la fosforilación oxidativa y el ciclo del ácido tricarboxílico (TCA), así como cambios epigenéticos (metilación del ADN) en los órganos vitales [38].

Se ha reportado que las proteínas no estructurales (NSPs) del SARS-CoV-2, más particularmente la NSP1, que se une a la sub­unidad ribosomal 40S de las células del huésped, causan la interrupción de la traducción del ARNm en las células del huésped [36]. También se ha descubierto que NSP1 bloquea el gen I inducible por ácido retinoico (RIG-I) y los genes estimulados por interferón (ISG), que son mediadores clave de la respuesta inmune innata del huésped en caso de infecciones virales [39]. Otra proteína del SARS-CoV-2, la NSP16, junto con la NSP10, protege al virus de la respuesta inmune innata del huésped mediante la metilación del extremo 5′ de los ARNm codificados por el virus (imitando así los ARNm celulares del huésped) [40].

Además de los mecanismos mencionados, recientemente se ha demostrado que la proteína spike del SARS-CoV-2 se mimetiza con un canal iónico de células epiteliales humanas, con lo que se dificultan sus funciones fisiológicas. Anand et al. [41] han demostrado que el sitio de escisión S1/S2 de la proteína «S» del SARS-CoV-2 tiene una sorprendente similitud en su secuencia proteica con el segmento del péptido que puede ser eliminado por la furina (FCS) en la subunidad α del canal de sodio epitelial humano (ENaC-α). El mimetismo de la proteína viral «S» con ENaC-α en las células huésped indica que el virus puede competir por la furina disponible en las células huésped infectadas y, por tanto, puede bloquear la activación proteolítica de ENaC-α. Se ha establecido que la ENaC-α interviene en el desarrollo del síndrome de dificultad respiratoria aguda (SDRA) mediado por la activación de las células inmunitarias y las citocinas/quimiocinas [42, 43], lo que indica que este mecanismo puede tener un papel en la patogénesis del SDRA en los pacientes graves de COVID-19.

## Desregulación del sistema renina-angiotensina-aldosterona (SRAA): Un eje clave para mantener la homeostasis fisiológica

**Fig. 4:**
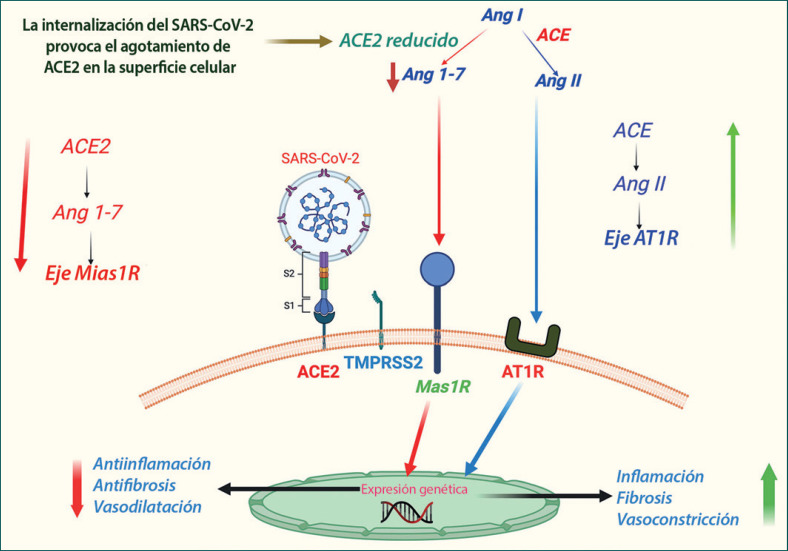
Esquema de la desregulación del SRAA mediada por la ACE2 en el COVID-19. El receptor de entrada a la célula huésped del SARS-CoV-2, ACE2, es un análogo de la ACE que participa en un paso clave en la regulación del SRAA: la conversión de Ang I en Ang II en el epitelio pulmonar. La Ang II actúa principalmente a través del receptor AT1. Alternativamente, la Ang II se metaboliza para producir angiotensina 1–7 (Ang 1–7), que actúa adicionalmente a través del Mas 1R. Fisiológicamente, el eje ACE/Ang II/AT1R se mantiene en equilibrio con el eje ACE2/Ang 1–7/Mas 1R. Al parecer, la unión del SARS-CoV-2 regula a la baja la señalización de la ACE2 y, en consecuencia, el eje ACE/Ang II/AT1R se impone, favoreciendo la constricción vascular, la inflamación en los tejidos y la fibrosis.

La desregulación del sistema renina-angiotensina-aldosterona (SRAA) ha sido un rasgo característico del COVID-19 [44]. El SRAA regula el equilibrio hemodinámico fisiológico que afecta a todos los órganos principales, principalmente el hígado, el pulmón, el corazón y el riñón [22], y también participa en el mantenimiento del equilibrio electrolítico y la resistencia vascular; por lo tanto, es un determinante crucial de la presión arterial sistémica y, en consecuencia, de la salud cardiovascular. La desregulación del SRAA inducida por el SARS-CoV-2 está mediada por su receptor de entrada en la célula huésped ACE2, un análogo de la ACE que realiza un paso clave en la regulación del SRAA: la conversión de angiotensina I en angiotensina II. El equilibrio ACE/ACE2 se considera un factor crucial para mantener el funcionamiento óptimo del SRAA. Aparentemente, la infección por SARS-CoV-2 regula a la baja la ACE2, pero no la ACE, y por lo tanto puede estar creando un desequilibrio de la relación fisiológica ACE/ACE2 [9, 17]. Por otra parte, la unión del SARS-CoV-2 provoca una regulación a la baja de la ACE2 en los componentes del SRAA, lo que puede inducir la activación y liberación de marcadores proinflamatorios que causan lesiones tisulares [42]. La desregulación del SRAA mediada por la ACE es un paso clave en la patogénesis del COVID-19 y contribuye significativamente a la mortalidad asociada con la comorbilidad, la trombosis vascular, la morbilidad específica de los órganos y las fallas multiorgánicas, que se discuten con detalle más adelante en las subsecciones relacionadas de este artículo. En la Figura 4 se resume una representación esquemática de las desregulaciones del SRAA mediadas por la ACE e inducidas por el virus en el COVID-19.

## Afectación multisistémica en el COVID-19: Comprensión de los mecanismos subyacentes

La amplia distribución en el cuerpo humano de los factores de entrada a las células huésped del SARS-CoV-2 indica que el virus puede infectar la mayoría de los órganos y tipos de tejidos. Inicialmente, se percibía que el principal órgano implicado en la patogénesis del COVID-19 era el pulmón; sin embargo, la acumulación de evidencias a lo largo del tiempo ha establecido que el COVID-19 es una enfermedad sistémica con manifestaciones extremadamente diversas [5] (Tabla 1). En muchos casos, los pacientes presentan sobre todo síntomas no-respiratorios que afectan a uno o varios órganos, incluido el cerebro [5]. Una revisión exhaustiva de la bibliografía sugiere que la afectación multisistémica en el COVID-19 puede explicarse en gran medida por la distribución generalizada de los receptores de entrada a las células huésped del SARS-CoV-2, la toxicidad viral directa, la respuesta inmunitaria desregulada del huésped, la participación del SRAA y la respuesta hiperinflamatoria sistémica contra la infección, además de la trombosis macro y microvascular. A continuación se mencionan evidencias empíricas recientes sobre todos estos aspectos, implicados en la fisiopatología del COVID-19 sobre sistemas fisiológicos clave.

### Sistema respiratorio

Los receptores de entrada celular del SARS-CoV-2 se expresan en múltiples tipos de células a lo largo del tracto respiratorio. En el tracto respiratorio superior, las células epiteliales ciliadas secretoras nasales coexpresan ACE2 y TMPRSS2 en abundancia [32]. En el tracto respiratorio inferior, ACE2 y TMPRSS2 se coexpresan de forma más extensa en los neumocitos de tipo 2 que en los de tipo 1; además, ambos receptores se expresan en las células caliciformes, de Clara, enteroendocrinas y bronquiales, así como en las células endoteliales de la vasculatura pulmonar [32, 33, 44]. Aunque los síntomas respiratorios leves pueden atribuirse a la infección del tracto respiratorio superior, el SARS o SDRA característico se produce cuando se infectan las células pulmonares, principalmente los neumocitos de tipo 2 y las células endoteliales vasculares pulmonares [45]. La infección de estas células puede provocar inflamación pulmonar grave, primero por la participación de los macrófagos residentes y luego por el reclutamiento de macrófagos periféricos y otras células inmunitarias, como neutrófilos y células T [20, 45]. La producción reducida de surfactantes (por parte de los neumocitos de tipo 2) y la consolidación de exudados acumulados provocan el colapso de los alveolos e inducen cambios neumónicos [46]. Si no se controla, la inflamación inicial puede provocar la liberación de más citocinas proinflamatorias y el reclutamiento de células inmunitarias periféricas, con lo que se produce un círculo vicioso que induce mayores lesiones tisulares [20]. La endotelitis vascular pulmonar y la posterior trombosis, principalmente de los microvasos, marcada por niveles sanguíneos elevados de productos de degradación de la fibrina (FDP), del dímero D y un mayor tiempo de protrombina (TP) también contribuyen en gran medida a la patología pulmonar [20, 45, 47].

### Sistemas cardiovascular y renal

Se sabe que los miocitos cardiacos, el endotelio de los vasos coronarios y los fibrocitos muestran una expresión significativa de ACE2 y de serina proteasas, principalmente TMPRSS2 [48]. Los síntomas cardiacos pueden deberse a la lesión miocárdica directa causada por el virus [49, 50]. Además, la desregulación del SRAA impulsada por el CoV-2 puede aumentar la incidencia de tromboembolismo, y los episodios hipertensivos pueden ser consecuencia de la constricción persistente de los vasos sistémicos y coronarios [51, 52]. Por otra parte, la inflamación de las arterias coronarias puede acelerar la formación de placas, provocando obstrucción y, por tanto, cambios isquémicos que conducen a la insuficiencia cardiaca [12, 53]. El desequilibrio electrolítico inducido por la desregulación del SRAA puede ser otro mecanismo que conduzca a enfermedades cardiacas; en particular, la hipopotasemia puede causar la hiperpolarización de los miocitos cardiacos y provocar arritmias [53]. La hipopotasemia en el COVID-19 también puede ser consecuencia de una lesión miocárdica directa mediada por el virus, que provoca la reducción del gasto cardiaco, activando así la excreción renal de iones de potasio mediada por la aldosterona [53, 54].

La afectación renal es muy indicativa en el COVID-19, teniendo en cuenta la significativa expresión del receptor de entrada a la célula huésped del SARS-CoV-2, ACE2, y de las proteasas asociadas, en particular TMPRSS2, en las células epiteliales de los túbulos renales y los podocitos de los glomérulos [33, 55]. En este sentido, se ha reportado afectación renal aguda y evidencia de lesión inducida por el virus, incluyendo la presencia de partículas virales en los túbulos renales en el examen histopatológico post mortem [34]. Sin embargo, la prevalencia de la afectación renal directa en el COVID-19 es baja si se compara con la de otros sistemas/órganos clave [56]. La lesión renal también puede ser secundaria al ARDS y a la sepsis inducida por la tormenta de citocinas. También puede deberse a la desregulación del SRAA mediada por la ACE2 o a múltiples causas iatrogénicas, como consecuencia del manejo en la unidad de cuidados intensivos o la ventilación mecánica y los efectos nefrotóxicos de los fármacos [57].

### Sistema digestivo

Se han observado síntomas digestivos prominentes en muchos pacientes con COVID-19 [6, 13], y se han detectado partículas de SARS-CoV-2 en las células, con evidencia de lesiones inflamatorias, en los tejidos gastrointestinales, en múltiples estudios post mortem [34, 58]. Los factores de entrada del SARS-CoV-2 en las células del huésped (ACE2 y TMPRSS2) se encuentran enriquecidos en el tejido intestinal [13], y se ha demostrado el éxito de la invasión viral en organoides intestinales humanos [59, 60] y en estudios de modelos animales, incluso en primates [61, 62].

Aunque actualmente hay suficientes evidencias de que el SARS-CoV-2 puede infectar el tracto gastrointestinal (TGI), todavía no se sabe bien cómo llega el virus a éste. La vía fecal-oral de entrada del virus es la explicación más plausible [62]. En algunos pacientes de COVID-19 se ha detectado la excreción de SARS-CoV-2 infeccioso en las heces, aunque no ha sido un hallazgo regular [62]. Es intrigante cómo el SARS-CoV-2 sobrevive a los extremos de pH en el medio del sistema digestivo (gástrico, 1,5–3,5; pancreático, 7,5; ácido biliar, 7–8) mientras atraviesa el TGI. Se sabe que el SARS-CoV-2 sobrevive en un amplio rango de valores de pH a temperatura ambiente (pH 3–10) [13]. Los ARN virus como el de la influenza A y B (cuando se ingieren) pueden sobrevivir a los extremos de pH y mantener su infectividad con ayuda de la capa de moco que recubre el tracto gastrointestinal, lo que permite su paso seguro e incluso su excreción en las heces [63]. Como las células mucosas son abundantes en todo el TGI, pueden contribuir al transporte y la supervivencia del SARS-CoV-2 [13].

Cabe destacar que la mucosa intestinal sana podría no favorecer la entrada del virus debido a la presencia de un sistema de barrera multicapa único, aunque la presencia de una condición inflamatoria previa que altere la barrera de la mucosa puede volverla permisiva [13, 64]. Además, una condición inflamatoria en el TGI puede favorecer la entrada del virus, al inducir la expresión de ACE2 en el epitelio de la mucosa [13]. Así, una condición inflamatoria intestinal previa, como la enfermedad inflamatoria intestinal (EII), puede aumentar la susceptibilidad a la infección por SARS-CoV-2 por vía fecal-oral [13]. La composición del microbioma intestinal del sujeto es otro factor importante que influye en la contractibilidad y la gravedad de los síntomas [65] (esto se analiza más a fondo en la subsección *Microbioma intestinal* en la sección *Factores del huésped que afectan la transmisibilidad, la gravedad y la evolución de los pacientes*).

Además de la vía fecal-oral, una ruta alternativa de entrada del virus a las células del TGI es a través de la microvasculatura tisular [13]. La evidencia actual indica que la sangre transporta y transmite el SARS-CoV-2, y el endotelio vascular expresa abundantemente la ACE2 y la TMPRSS2 [45, 66]. La inflamación y la lesión tisular inducidas por la infección viral, incluida la ruptura de los complejos de unión intercelular, pueden hacer que los pequeños vasos sean permeables para el paso del virus [67].

Además del TGI, se han observado lesiones tisulares en otros componentes del sistema digestivo, como el hígado, las vías biliares y el páncreas [34, 68]. La elevación de las enzimas hepáticas ha sido un hallazgo frecuente en los casos graves de COVID-19 [68]. Paradójicamente, no ha habido evidencias claras de una invasión viral directa al tejido hepático [34]; sin embargo, los estudios en modelos de organoides de tejidos humanos han demostrado que el SARS-CoV-2 puede infectar los hepatocitos, así como los colangiocitos del epitelio ductal biliar [35]. Cabe destacar que los estudios proteómicos y transcriptómicos sugieren que la expresión del receptor de entrada de la célula huésped viral ACE2 se reduce principalmente a los colangiocitos [69]. Al observar la limitada expresión de ACE2 en el tejido hepático, queda la posibilidad de que cualquier deterioro hepático en el COVID-19 pueda deberse principalmente no a una lesión vírica directa, sino a razones indirectas, como la hiperinflamación sistémica, la respuesta inmunitaria desregulada y la trombosis de los microvasos. La afectación del páncreas en el COVID-19 es intrigante, porque se ha observado en los pacientes una grave alteración de la glicemia, incluida la nueva aparición de diabetes (como se analiza más adelante en la subsección *Comorbilidades*) [70, 71]; sin embargo, aún no hay evidencias claras de que el COVID-19 pueda hacer esto por sí solo (revisado en [72]). Aunque ha habido indicios vagos en estudios proteómicos y transcriptómicos de alto rendimiento sobre la secreción de ACE2 por los componentes pancreáticos [13], los estudios de secuenciación de ARN unicelular mostraron claramente una expresión significativa de ACE2 en las células beta secretoras de insulina, y se demostró que el SARS-CoV-2 es capaz de infectar las células endocrinas (alfa y beta) en organoides pancreáticos humanos [35].

### Sistema nervioso

Se han reportado síntomas neurológicos, leves en la mayoría de los casos, como cefalea, náuseas, vómito, mareo y pérdida de los sentidos (olfato y gusto), y en ciertos casos signos graves como ataxia, convulsiones, alteración de la conciencia, accidente cerebrovascular isquémico o hemorrágico, encefalomielitis aguda diseminada (ADEM), meningitis, encefalitis y, raramente, variantes del síndrome de Guillain-Barré (síndrome de Miller Fisher y polineuritis craneal) [8, 73, 74, 75, 76], así como la aparición de síntomas psicóticos [77] en el COVID-19. Estudios de autopsia en fallecidos por COVID-19 han mostrado lesiones cerebrales generalizadas (que reflejan sobre todo una lesión hipóxico-isquémica aguda) [78]. Aunque es poco frecuente, el ARN viral también se detecta en el tejido cerebral (78) y en el líquido cefalorraquídeo (LCR) [74, 79] de los fallecidos por COVID-19.

La forma como el SARS-CoV-2 entra en el sistema nervioso central y media en la patogénesis de los síntomas neurológicos en pacientes con COVID-19 empieza a explicarse a la luz de nuevos datos [80]. La ruta más probable de diseminación viral al cerebro es la propagación transneuronal a través de los nervios olfatorios. También es posible la vía hematógena tras atravesar la barrera hematoencefálica (BHE) [67]. Los estudios observaron una expresión significativa de ACE2 y TMPRSS2 en las células del epitelio olfativo y la mielina (oligodendrocitos) y el endotelio neurovascular en humanos [80]. Además, diversos estudios de modelos en ratones transgénicos, que expresan ACE2 humana, han demostrado la propagación intracraneal del SARS-CoV-2 a otras partes del cerebro a través de la vía olfativa tras la inoculación intranasal [80]. El conjunto de evidencias favorece fuertemente el potencial neuroinvasivo del SARS-CoV-2. Además, la nueva aparición de síntomas psicóticos en algunos pacientes del COVID-19 [77] indica que se produce una patología sináptica en las regiones cerebrales asociadas con las funciones ejecutivas.

Los estudios sugieren que los síntomas neurológicos (incluida la pérdida del olfato y el gusto) pueden surgir debido al efecto neuropático directo del virus. Alternativamente, podría tratarse de un efecto indirecto de la neuroinflamación inducida por citocinas o de un efecto mediado por células inmunitarias [81, 82] sobre las neuronas (o las células gliales) o las células endoteliales de la microvasculatura cerebral, que inducen apoptosis celular y aumentan la permeabilidad vascular y el edema en el tejido cerebral relacionado.

La mediación de la infección por SARS-CoV-2 en el cerebro por el recientemente propuesto receptor alternativo NRP1 es muy plausible. Curiosamente, el NRP1 se expresa de forma significativa en el cerebro humano, incluidas las neuronas olfativas. La prueba del concepto de la entrada del SARS-CoV-2 en el cerebro mediada por el NRP1 procede de un estudio reciente de Cantuti-Castelvetri et al. [29] en el que se demuestra la presencia de la proteína spike del SARS-CoV-2 en las neuronas que expresan NRP1 y en las células endoteliales de capilares y vasos de tamaño medio del bulbo y el tracto olfativo en las muestras de autopsia del cerebro de los fallecidos por COVID-19. Los autores también demuestran en ratones, tras la administración intranasal, la entrega dependiente de NRP1 de nanopartículas del tamaño del virus (80 nm de diámetro) al epitelio olfativo y a las células neuronales del bulbo olfativo y del sistema nervioso central (corteza), lo que significa que la vía olfativa es una ruta de entrada del SARS-CoV-2 en el cerebro [29].

Los síntomas neurológicos también pueden surgir por otros motivos, como la encefalopatía metabólica derivada de la disfunción de órganos vitales (como el pulmón, el hígado y el riñón) [7] o un mayor riesgo de trombosis neurovascular en pacientes con COVID-19 grave [83], comorbilidades asociadas o patologías neurovasculares relacionadas con la edad.

### Otros sistemas y tipos de tejido

El COVID-19 no sólo afecta los sistemas fisiológicos clave (mencionados anteriormente), sino también casi todos los demás sistemas [5], incluidos los órganos sensoriales, es decir, los ojos [11] y los oídos [10], además de los tegumentos, es decir, la piel, el pelo y las uñas [15, 16]. La afectación patológica de la mayoría de estos sistemas/tipos de tejido puede predecirse con base en la expresión de los factores de entrada a las células huésped del virus [31] (Figura 2). Los mecanismos responsables de la lesión del tejido infectado por el SARS-CoV-2 pueden ser múltiples, a saber, una lesión directa causada por la citotoxicidad vírica [1], disfunción endotelial mediada por el receptor de entrada de la célula huésped vírica ACE2 y la consiguiente trombosis vascular [45], o daño inflamatorio debido a una respuesta inmunitaria excesiva contra la infección [20], o puede deberse a un mecanismo independiente del virus, como ciertas inmunopatologías específicas de los tejidos que actualmente no están bien explicadas [84].

## Respuesta inmunitaria del huésped a la infección por el SARS-CoV-2

La infección por el SARS-CoV-2 provoca respuestas inmunitarias diferentes en los distintos individuos, las cuales determinan la gravedad de los síntomas [85]. Algunos individuos se recuperan con los síntomas más leves o permanecen completamente asintomáticos; en cambio, en otros, la infección vírica provoca manifestaciones graves de la enfermedad que dan lugar al SDRA y a la falla multiorgánica [20]. Al igual que en otras infecciones víricas, la defensa innata mediada por citocinas es la primera respuesta inmunitaria en los individuos infectados. Una forma peculiar de estado hiperinflamatorio sistémico, caracterizado por niveles muy elevados de citocinas proinflamatorias, conocido como «tormenta de citocinas» (TC) se observa comúnmente en pacientes con COVID-19 grave [20]. Cabe destacar que la TC no es exclusiva del COVID-19, y ha sido un hallazgo característico en la fase grave de muchas otras enfermedades víricas respiratorias, como el SARS, el MERS y la influenza [86]. Sin embargo, la TC inducida por el SARS-CoV-2 es diferente que la de otros virus respiratorios, porque el SARS-CoV-2 no induce necesariamente una firma común de citocinas, como la interleucina (IL)-2, la IL-10, la IL-4 o la IL-5 [87]. En el COVID-19, la TC se caracteriza por un conjunto particular de citocinas muy aumentadas en el suero de los pacientes, como la IL-2, la IL-7, el factor estimulante de colonias de granulocitos y macrófagos (GM-CSF), el factor estimulante de colonias de granulocitos (G-CSF), la proteína 10 inducida por interferón gamma (IP10), la proteína inflamatoria de macrófagos 1-α (MIP1-α), la proteína quimioatrayente de monocitos 1 (MCP-1), el factor de necrosis tumoral α (TNFα) y el interferón γ (IFN-γ). Las concentraciones circulantes del ligando 10 de la quimiocina (motivo C-X-C) (CXCL10), del ligando 2 de la quimiocina (motivo C-C) (CCL2), de la IL-2R, de la IL-6, del TNFα, de la proteína C-reactiva (PCR) y de la ferritina son significativamente más elevadas en las personas que deben ingresar en la unidad de cuidados intensivos (UCI) [87]. Además, la TC se dirige especialmente a la desregulación de la respuesta del IFN tipo I y sus citocinas descendentes [87].

**Fig. 5:**
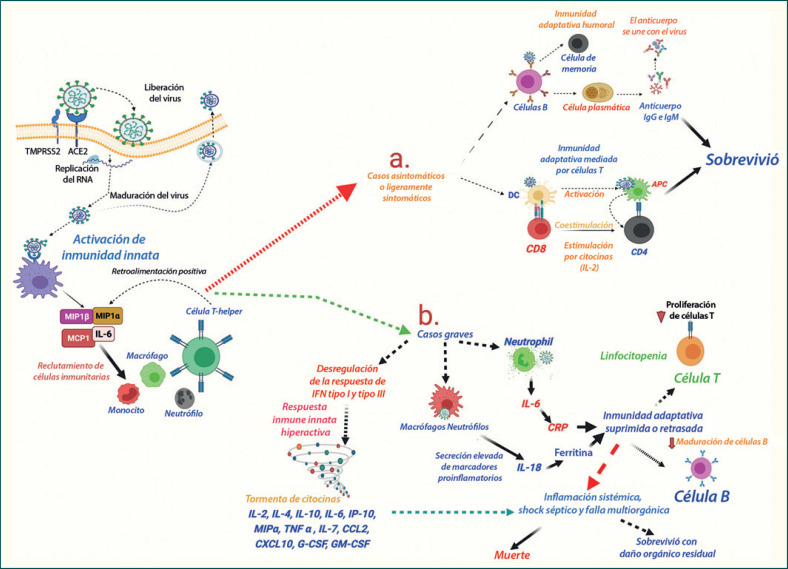
Descripción esquemática de las respuestas inmunitarias en los casos asintomáticos, ligeramente sintomáticos y graves de COVID-19. La invasión del SARS-CoV-2 mediada por el receptor de entrada a la célula huésped y las proteasas asociadas con la entrada conduce a la activación de la respuesta inmunitaria innata y al reclutamiento de células inmunitarias circulantes en el epitelio pulmonar. Además, la respuesta inmunológica es diferente en casos asintomáticos/ligeramente sintomáticos y graves de pacientes con COVID-19: (**a**) en los casos asintomáticos y levemente sintomáticos, una activación óptima de la respuesta inmunitaria adaptativa humoral y mediada por células T conduce a la curación de los pacientes; (**b**) en los casos graves, se observa una respuesta inmunitaria innata hiperactiva que conduce a una tormenta de citocinas y, en consecuencia, a la muerte de las células T y a una respuesta humoral mediada por células B retrasada o suprimida, que da lugar a resultados muy desfavorables para los pacientes.

Los investigadores de todo el mundo se esfuerzan por comprender las razones exactas de la mayor respuesta inmunitaria innata observada en los pacientes con COVID-19. Una revisión de la literatura existente sugiere que puede explicarse en parte por los hechos conocidos sobre las interacciones únicas entre el huésped y los virus respiratorios (revisado en [20]). La primera defensa del huésped contra una infección vírica está marcada por un aumento de la molécula clave de la respuesta inmunitaria innata: el IFNα [20]. Al igual que en el SARS y el MERS [88], se ha observado una respuesta retardada del IFNα en el COVID-19, lo que indica que se trata de un mecanismo mediado por el virus para facilitar su entrada en la célula. Una respuesta inmune retardada facilitaría la entrada del virus en el epitelio pulmonar y eso, a su vez, llevaría a una intensa respuesta inflamatoria causada por el creciente reclutamiento del amplio repertorio de células inmunes innatas [89]. Curiosamente, a diferencia de otros virus respiratorios, como el SARS-CoV, el MERS-CoV y el virus de la influenza tipo A (IVA), el SARS-CoV-2 parece utilizar una estrategia distintiva [90]. El SARS-CoV-1, el MERS-CoV y el IVA provocan una atenuación completa de la respuesta inmunitaria innata mediada por el IFN o las quimiocinas (citocinas proinflamatorias con propiedades quimioatrayentes) en el momento de la infección. Por el contrario, el SARS-CoV-2 mostró una respuesta atenuada de IFN (tipo I, y también tipo III), pero paradójicamente elevó mucho las quimiocinas [89] (Figura 5). La atenuación de la respuesta temprana del IFN contra la infección por el SARS-CoV-2 está mediada por la proteína viral ORF3b [90]. Esta dicotomía única en la respuesta inmunitaria innata del huésped en el COVID-19 puede promover la invasión viral del epitelio respiratorio (y de las células epiteliales en otros sitios) y facilitar la replicación viral. A su vez, una carga viral elevada en las células epiteliales infectadas del huésped puede inducir una lesión tisular y, como consecuencia, un aumento de la secreción de quimiocinas y del reclutamiento de células inmunitarias circulantes (Figura 5). Una respuesta inmunitaria innata hiperactiva dificultaría, paradójicamente, la eliminación del virus y promovería una mayor replicación viral [1].

Una respuesta inmunitaria adaptativa mediada por células T y humoral óptima es habitual en los casos asintomáticos y sintomáticos leves. Por el contrario, en pacientes con COVID-19 grave, en el periodo inicial se observa una respuesta inmunitaria adaptativa mediada por células T protectora subumbral y retardada en los pacientes sintomáticos. Sin embargo, los supervivientes mostraron una respuesta inmune adaptativa robusta y duradera [91] (revisado en [92]).

Curiosamente, se ha observado que las células Th1, un tipo de células T CD4 activadas, y las células Th17 similares a las células de memoria del tejido pulmonar (Trm 17), caracterizadas por una expresión potencialmente patógena de las citocinas IL-17A y GM-CSF, son más elevadas en los pacientes con enfermedad grave que en quienes padecen una enfermedad moderada [93]. Es interesante observar que el GM-CSF se elevó de forma distintiva sólo en los casos graves de COVID-19 y no en los de influenza cuando se compararon ambas condiciones [94].

La linfocitopenia, en particular de células T y más intensamente de células T CD8, es una observación común independientemente del estadio y la gravedad de los casos de COVID-19 [22]. Sin embargo, la linfocitopenia se correlaciona con la gravedad de los síntomas, y un número muy bajo de células T predice una mala evolución de los pacientes [95, 96]. Por el contrario, en la mayoría de los pacientes con síntomas leves o moderados y con un recuento estable de linfocitos, los anticuerpos neutralizantes [inmunoglobulina G (IgG)] para el spike viral u otras proteínas aparecen casi siempre entre los días, 18 y 21, y el pronóstico es bastante bueno. Las razones moleculares de la muerte de las células T en el COVID-19 son poco conocidas [97]. Cabe destacar que las células T muestran una menor expresión de los receptores de entrada del SARS-CoV-2, por lo que la muerte de las células T mediada por el virus en los individuos infectados puede producirse por algún otro mecanismo y no por la señalización del receptor viral [85]. La atrofia y la lesión de los tejidos linfoides humanos, como el bazo y los ganglios linfáticos, mediadas por el SARS-CoV-2 también se han evidenciado en estudios recientes [58, 98, 99]. El análisis de los parámetros de laboratorio en pacientes con COVID-19 sugiere que los altos niveles de marcadores proinflamatorios, o la tormenta de citocinas podrían explicar la eliminación de las células T [100]. Los niveles de TNF-α, IL-6, IL-8 e IL-10 se encuentran significativamente aumentados y se correlacionan negativamente con los recuentos de células T en el COVID-19 grave [100]. Es bien sabido que los niveles séricos elevados de TNF-α y otras citocinas inducen apoptosis de las células T en las enfermedades inflamatorias, incluidos el SARS y el MERS [101].

De acuerdo con la revisión de la evidencia disponible (discutida anteriormente), una mejor replicación en las células del huésped que resulta en una mayor carga viral y la desregulación mediada por el virus de la respuesta inmune innata y adaptativa del huésped parece ser el mecanismo más importante detrás de la mayor virulencia y riesgo de mortalidad del SARS-CoV-2 con respecto a la influenza y otros BCoVs (SARS-CoV-1 y MERS-CoV). Por lo tanto, junto con una reducción de la replicación viral mediante fármacos antivirales, el manejo de la respuesta inmunitaria desregulada del huésped, específicamente la innata, mediante el uso de inmunomoduladores, citocinas antiinflamatorias y anticuerpos dirigidos a citocinas o las vías proinflamatorias, constituye una importante estrategia terapéutica en el COVID-19 (revisado en [20]). Cabe destacar que, además de ser resultado de la desregulación inducida por el SARS-CoV-2, la respuesta inmunitaria innata hiperactiva puede deberse a factores intrínsecos del sujeto [102, 103]. Concretamente, en algunos adultos jóvenes y sanos sin comorbilidad evidente, el desarrollo de COVID-19 grave y la posterior mortalidad indican claramente la presencia de una vulnerabilidad específica del sujeto [103]. Es probable que estos individuos tengan una predisposición genética o inmunofenotípica para desarrollar la enfermedad grave [102, 103]. Discutimos esta cuestión con más detalle en la sección *Factores del huésped que afectan la transmisibilidad, la gravedad y la evolución de los pacientes* (subsección *Factores genéticos e inmunofenotípicos*).

## Fisiopatología de la trombosis vascular y la falla multiorgánica

La trombosis de los macrovasos y microvasos en los órganos, especialmente en la vasculatura pulmonar, ha sido una manifestación destacada del COVID-19 grave. La trombosis vascular se ha relacionado con la génesis de fallas multiorgánicas y se ha asociado con desenlaces muy desfavorables de la enfermedad, incluyendo una mayor mortalidad. Se han sugerido múltiples razones para la etiología de la trombosis vascular en el COVID-19, como la toxicidad de las proteínas víricas, niveles elevados de marcadores proinflamatorios y la tormenta de citocinas, el impacto protrombótico de la enfermedad grave y causas iatrogénicas [47, 104, 105]. Sin embargo, un mecanismo mediado por la unión del SARS-CoV-2 al receptor ACE2 parece encontrarse en la raíz de todos estos mecanismos [45, 105, 106]. Los factores de entrada del SARS-CoV-2 en la célula huésped, ACE2 y TMPRSS2, se coexpresan en las células endoteliales de los vasos sanguíneos humanos y la microvasculatura [45], así como en los componentes de las células sanguíneas, en particular las plaquetas [106]. La regulación a la baja de ACE2 inducida por la unión del SARS-CoV-2 puede inducir la activación y liberación de marcadores proinflamatorios y, por tanto, puede causar lesiones en el endotelio vascular [45, 107, 108]. Además, una inversión del cociente ACE/ACE2 en la disfunción del endotelio vascular también puede inducir trombosis [45]. Se sabe que un valor más bajo del cociente ACE/ACE2 en el endotelio vascular evita la activación de la cascada protrombótica, al catalizar la degradación de la angiotensina I (Ang I) a angiotensina 1–9 inactiva y de la angiotensina II (Ang II) a angiotensina 1–7, con funciones antiproliferativas, antifibróticas y vasodilatadoras por medio de los receptores Mas acoplados a proteínas G [45, 105]. Por el contrario, un cociente ACE/ACE2 más elevado permite una mayor conversión de Ang I en Ang II y la unión de esta última a sus receptores de tipo 1 (AT1), por lo que puede inducir vasoconstricción, inflamación y fibrosis, y finalmente trombosis vascular [45, 105]. Además, la regulación a la baja de la ACE2 mediada por el SARS-CoV-2 en el endotelio vascular puede activar la vía de la calicreína-bradiquinina, induciendo la agregación plaquetaria y la filtración de los vasos, que puede agravar aún más los episodios trombóticos [45]. Alternativamente, la unión directa del SARS-CoV-2 a la ACE2 expresada en las plaquetas también puede inducir agregación plaquetaria y la consiguiente trombosis [106].

Es plausible que la activación del «sistema del complemento» del huésped por la toxicidad de las proteínas víricas y la respuesta inflamatoria sistémica inducida por las citocinas pueda contribuir significativamente a la patogénesis de la trombosis vascular [20]. Gao et al. [109] demostraron recientemente en ratones que las proteínas N de los BCoV (SARS-CoV, MERS-CoV y SARS-CoV-2) se unen a la lectina serina proteasa 2 de unión a mananos (MASP-2) (una serina proteasa clave en la activación del complemento), lo que provoca una activación aberrante del sistema del complemento del huésped y, en consecuencia, trombosis microvascular y una lesión pulmonar inflamatoria agravada. De manera interesante, la activación viral del sistema del complemento se revirtió con la aplicación de anticuerpos contra MASP-2 [109].

En los vasos pulmonares, principalmente en los microvasos, se observa con frecuencia la neutrofilia y la formación de trampas extracelulares de neutrófilos (NETs) en casos graves. La activación de la NETosis conduce a un aumento de la concentración de especies reactivas de oxígeno (ROS) intracelulares en los neutrófilos, lo que induce disfunción endotelial y activa las vías de coagulación (tanto extrínsecas como intrínsecas), por lo que puede sumarse al evento de trombosis vascular en curso. La hiperviscosidad inducida por la hipoxia y la regulación al alza de la vía de señalización HIF-1α (factor inducible por hipoxia 1 alfa) pueden ser factores adicionales que contribuyan a la trombosis [5].

La trombosis vascular, sumada a la toxicidad directa de las proteínas víricas en el tejido infectado y al desequilibrio hemodinámico mediado por la desregulación del SRAA, la hiperinflamación sistémica y el síndrome de choque tóxico derivado de la tormenta de citocinas, puede culminar en la falla multiorgánica [105, 110, 111]. La presencia de comorbilidades y la vulnerabilidad específica del huésped ante los síntomas graves de la enfermedad pueden contribuir aún más a la patogénesis de la falla multiorgánica [21, 112].

**Fig. 6:**
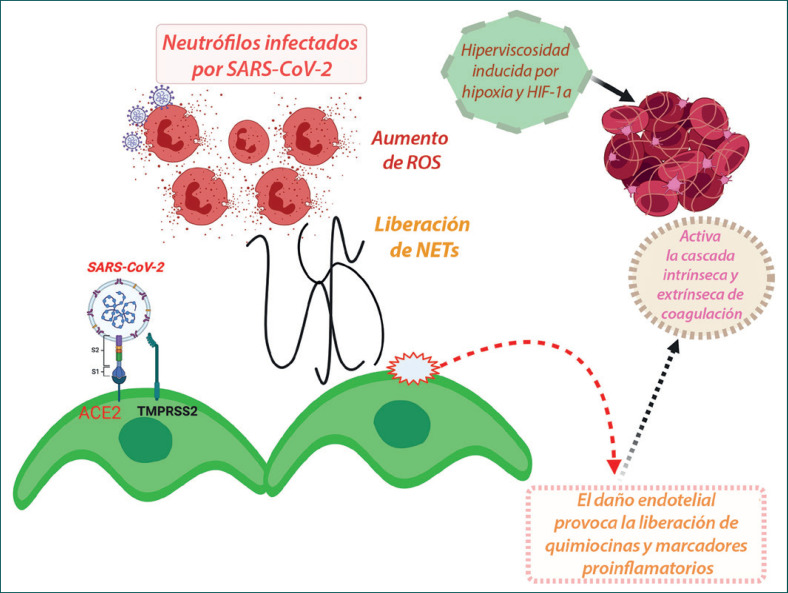
Esquema de la disfunción del endotelio vascular mediada por la ACE2, que provoca trombosis en los pacientes de COVID-19. La unión del SARS-CoV-2 con el receptor ACE2 expresado en la superficie de la célula endotelial vascular conduce a la internalización y replicación del virus dentro de la célula y, en consecuencia, a la disfunción endotelial que activa la cascada protrombótica. Además, la unión del SARS-CoV-2 induce la regulación a la baja de ACE2, lo que da lugar a desequilibrios en el cociente ACE/ACE2, y a la desregulación del SRAA, favoreciendo la protrombosis. Ambos mecanismos mencionados inducen también, en consecuencia, la activación y agregación de las plaquetas, desembocando en conjunto en la trombosis intravascular. Además, las NETs que provocan un aumento de las concentraciones de ROS intracelulares en los neutrófilos inducen la disfunción endotelial vascular y la activación de las vías de coagulación. Además, la hiperviscosidad inducida por la hipoxia y la regulación al alza de la vía de señalización HIF-1α pueden contribuir a la trombosis vascular.

En la Figura 6 se resume una descripción esquemática de la disfunción del endotelio vascular mediada por la ACE2, en la que interviene el SRAA, y las consiguientes incidencias trombóticas y el desarrollo de fallas multiorgánicas en los pacientes con COVID-19.

## Factores del huésped que afectan la transmisibilidad, la gravedad y la evolución de los pacientes

Algunos grupos de población específicos, principalmente varones, ancianos y personas con comorbilidades, se han visto afectados de forma más agresiva por el COVID-19 [21]. Las razones por las que la enfermedad afecta más a estos grupos de población se están descubriendo gradualmente. La evidencia científica disponible señala los múltiples factores intrínsecos al huésped que son responsables de la mala evolución en algunos individuos [21]. En esta sección discutiremos brevemente la evidencia empírica existente sobre la base patológica de la implicación de los principales factores intrínsecos del huésped, que se ha descubierto influyen significativamente en la transmisibilidad, la gravedad y el desenlace de los pacientes de COVID-19.

### Edad

En el COVID-19 es común observar una enfermedad más grave y una mayor mortalidad en individuos con más de 50 años, sobre todo en ancianos [113, 114]. Se han sugerido varios mecanismos biológicos para ello [115]. La inmunosenescencia en los individuos de edad avanzada podría ser la razón principal (116). La disponibilidad de células T naive, el cociente de células T CD4/CD8 y el número de células T reguladoras (Treg) disminuye con el envejecimiento [117]. Es plausible que la inmunosenescencia comprometa la respuesta contra un nuevo patógeno, como el SARS-CoV-2 [118]. Asimismo, la respuesta inflamatoria protectora empeora con el envejecimiento [116]. Por otra parte, las personas de edad avanzada tienen más probabilidades de padecer comorbilidades graves [115].

En cambio, en el grupo de edad pediátrica se ha reportado un menor número de casos y una mortalidad reducida. Sorprendentemente, se ha reportado un raro síndrome inflamatorio multisistémico en niños (MIS-C) en todo el mundo en este grupo de edad [119]. El MIS-C se presenta entre 4 y 6 semanas después de la infección por el SARS-CoV-2 en forma de fiebre alta y disfunción orgánica, y los pacientes muestran marcadores de inflamación muy elevados. La patogénesis de la MIS-C aún no se conoce con claridad. Tiene características clínicas que se superponen con la enfermedad de Kawasaki, lo que sugiere que se trata de una vasculitis de etiología autoinmune [120].

### Sexo

Se ha observado que la gravedad de la enfermedad y la mortalidad son significativamente mayores en los hombres que en las mujeres [121]. Un desglose de los datos globales actuales por sexo muestra que por cada 10 mujeres, respectivamente, 10 hombres están infectados, 12 son hospitalizados, 17 ingresan en la UCI y 13 mueren (https://globalhealth5050.org, con fecha 08/10/2021) [121].

La base causal por la que los hombres se ven más afectados en el COVID-19 aún no se comprende bien. Se han sugerido varias razones para ello (revisado en [122]). Se han propuesto como razones biológicas de la mala evolución de los hombres una mayor expresión de los factores de entrada del SARS-CoV-2 en las células huésped, ACE2 y TMPRSS2, en los órganos reproductores de los hombres [123, 124, 125] y la regulación androgénica de la TMPRSS2 [126]. Por el contrario, la vinculación con el cromosoma X y la regulación mediada por estrógenos de múltiples genes de respuesta inmunitaria, incluidos el IFN tipo 1 y el sensor viral TLR-7, podrían ser las razones principales por las que el pronóstico es comparativamente mejor en mujeres [127, 128].

### Comorbilidades

Las comorbilidades son los factores del huésped que más contribuyen a la gravedad del COVID-19. Los factores que más han contribuido al COVID-19 han sido, respectivamente, las enfermedades cardiovasculares, la diabetes, las enfermedades respiratorias crónicas, la hipertensión y la tejido tejido (revisado en [129]).

La hipertensión y la obesidad pueden predecir de forma independiente la evolución de los pacientes con COVID-19 [130, 131], y la diabetes preexistente puede incrementar el riesgo de padecer una enfermedad grave/crítica y la mortalidad intrahospitalaria en aproximadamente 2 y 3 veces, respectivamente [129, 132]. Las razones de la mayor vulnerabilidad al COVID-19 en presencia de comorbilidades aún no se comprenden bien. Es plausible que el daño residual y la desregulación de la fisiología pulmonar, y también de los demás órganos vitales, en los pacientes con comorbilidades se sumen a la patología inducida por el COVID-19 [129]. La desregulación del SRAA mediada por el receptor de entrada de la célula huésped del SARS-CoV-2, ACE2, también puede intervenir en ello [45].

La obesidad establece un estado de inflamación sistémica crónica y también cambia el fenotipo de las células inmunitarias de antiinflamatorias a proinflamatorias (células T CD4 de Th2 a Th1, y macrófagos de M2 a M1) e induce un aumento de la secreción de adipocitocinas proinflamatorias, como la leptina, y una disminución de la secreción de adipoquinas antiinflamatorias, como la adiponectina, lo que favorece una respuesta inflamatoria grave contra cualquier nueva infección [133]. Por lo tanto, la obesidad puede agravar la patología del COVID-19 al inducir una respuesta inflamatoria temprana y excesiva contra la infección viral, como las tormentas de citocinas [134].

En estudios recientes se han observado cambios en la glucemia, incluida la aparición de diabetes y mayores complicaciones en algunos pacientes con COVID-19 [72, 135]. El aumento de la mortalidad relacionado con complicaciones diabéticas ha sido frecuente en pacientes con COVID-19 [132]. El SARS-CoV-2 puede invadir las células de los islotes pancreáticos productoras de insulina [35]. Además, la regulación a la baja mediada por ACE2 del cotransportador 1 de sodio-glucosa (SGLT1) en el epitelio intestinal previene la hiperglucemia en modelos de diabetes mellitus en ratas [13]. La regulación a la baja de la expresión de ACE2 mediada por el SARS-CoV-2 puede conducir a la regulación al alza de SGLT1, precipitando así la hiperglucemia [13]. Además del intestino, el SGLT1 se expresa en otros tejidos humanos, como el túbulo proximal del riñón, el corazón y el hígado (proteinatlas.org/ENSG00000100170-SLC5A1/tissue). Por lo tanto, una invasión de las células de los islotes pancreáticos mediada por ACE2 y la desregulación de SGLT1 en el epitelio intestinal pueden ser mecanismos plausibles para la aparición de diabetes en los pacientes de COVID-19.

### Factores genéticos e inmunofenotípicos

Se conocen variantes polimórficas para el receptor de entrada a la célula huésped del SARS-CoV-2, ACE2, y las proteasas del huésped asociadas, como TMPRSS2 [136, 137] y furina [138]. Es posible que algunas de estas variantes polimórficas de los receptores del SARS-CoV-2 sean más comunes en las personas con una vulnerabilidad baja o alta para infectarse, o bien que afecten la gravedad de los síntomas y la mortalidad del COVID-19 [137].

Algunos estudios sugieren que la existencia de mutaciones en los sensores víricos del huésped y en los genes de la respuesta inmune podría aumentar la vulnerabilidad para el desarrollo de COVID-19 grave. Se ha descubierto que una mutación del receptor tipo Toll-7 (*TLR-7*) − un sensor viral (también para los coronavirus) en las células del huésped − está asociada con el COVID-19 grave [102]. Otro estudio ha reportado que al menos 3.5% de los pacientes con neumonía por COVID-19 potencialmente mortal tenían mutaciones conocidas en los genes de la respuesta inmunitaria, como el factor regulador de interferón 7 (*IRF7*) y el receptor de interferón (IFN)-alfa 1 y 2 (*IFNAR1* y -*2*), *TLR3*, la molécula adaptadora 1 que contiene el dominio TIR (*TICAM1*), la quinasa de unión a TANK 1 (*TBK1*) y el factor regulador de IFN 3 (*IRF3*) [139].

Se han encontrado dos regiones genómicas especialmente asociadas con el COVID-19 grave: una región en el cromosoma 3 (locus 3p31.21) que contiene seis genes y otra en el cromosoma 9 (locus 9q34.2) que representa los grupos sanguíneos ABO [21, 140]. Curiosamente, las variantes genéticas del cromosoma 3 (45.859.651–45.909.024 (hg19)) se introdujeron en la población humana mediante un flujo genético procedente de homínidos arcaicos no-*Homo sapiens:* los neandertales [141]. Se han reportado asociaciones en todo el genoma en múltiples loci cromosómicos además de los 3 y 9, como en la cr12q24.13 (rs10735079) en un grupo de genes que codifican activadores de enzimas de restricción antivirales (*OAS1, OAS2, OAS3*), en la cr19p13 2 (rs2109069) cerca del gen que codifica la tirosina quinasa 2 (*TYK2*), en el chr19p13.3 (rs2109069) en el gen que codifica la dipeptidil peptidasa 9 (*DPP9*), y en el chr21q22.1 (rs2236757) en el gen *IFNAR2* [103, 140]. Por el contrario, un estudio reciente descubrió que un haplotipo de 75 kb en el cromosoma 12 (pares de bases 113.350.796 a 113.425.679, rs1156361) se asociaba con una reducción de ∼22% en el riesgo relativo de desarrollar COVID-19 grave [142]. Se sugirió que los individuos con variantes del antígeno leucocitario humano (HLA), B*46:01 y B*15:03, respectivamente, eran más y menos propensos al SARS-CoV-2 y al COVID-19 grave [143].

El papel de los mecanismos epigenéticos en la respuesta inmunitaria del huésped en el COVID-19 se demostró en un reciente estudio in vitro en el que se utilizaron tamices CRISPR de edición del genoma (repeticiones palindrómicas cortas agrupadas y regularmente interespaciadas) en células Vero-E6. Los autores identificaron las moléculas reguladoras epigenéticas High Mobility Group Box 1 (HMGB1) y el complejo de remodelación de la cromatina SWItch/Sucrose Non-Fermentable (SWI/SNF), críticas para la muerte de las células huésped inducida por coronavirus, incluido el SARS-CoV-2 [144].

Se descubrió que las personas del grupo sanguíneo O tenían una probabilidad ligeramente menor de contraer COVID-19, así como una protección relativa contra el desarrollo de síntomas graves y la muerte en comparación con las personas de los grupos sanguíneos A, B y AB [145, 146, 147]. Cabe destacar que en un estudio de casos y controles muy reciente, en el que se incluyó un número elevado de pacientes con COVID-19 (más de 11.000 casos positivos), no se encontraron riesgos aumentados en las personas con grupos sanguíneos A, B y AB, ni protección alguna para el grupo sanguíneo O contra la infección, la hospitalización, la gravedad de la enfermedad ni su evolución [148].

La autoinmunidad también ha sido un factor en la patogénesis de los síntomas graves en el COVID-19. En un estudio se observó que al menos 10.2% de los pacientes con 25 a 87 años tenían autoanticuerpos contra el IFN tipo I, y de ellos 95 (94%) eran hombres. Se ha observado otro caso de generación de autoanticuerpos en pacientes con COVID-19 contra los fosfolípidos de la célula huésped [149]. La presencia de anticuerpos antifosfolípidos (aPL) protrombóticos en el suero de los pacientes con COVID-19 también podría ser una razón de la mayor incidencia de trombos en algunos pacientes [150, 151]. Por el contrario, un estudio de Borghi et al. [152] encontró una baja prevalencia de aPL en los pacientes con COVID-19 y ninguna asociación entre la trombosis y los aPL.

### Microbioma intestinal

Se afirma que el microbioma intestinal desempeña un papel importante en la gravedad de la enfermedad y en la evolución de los pacientes con COVID-19, porque refuerza la inmunidad local de la mucosa contra las invasiones virales [153]. Estudios recientes han demostrado que los pacientes con COVID-19 tienen un microbioma diferente al de los controles, enriquecido con los patógenos oportunistas, y el agotamiento de los comensales beneficiosos mostró correlación con la gravedad de los síntomas [65, 154]. Estos estudios han indicado además que la composición del microbioma intestinal puede influir en la producción de citocinas inflamatorias inducida por el SARS-CoV-2 y, por consiguiente, en la aparición de la tormenta de citocinas [65, 153, 155].

### Inmunidad cruzada y protección contra la enfermedad grave

Se encontraron células T y anticuerpos reactivos al SARS-CoV-2 en muchos individuos sin exposición previa [156, 157], lo que indica que las infecciones previas con otros CoVs podrían haber causado esto. Las infecciones respiratorias en humanos por CoVs, especialmente los que causan el resfriado común, son comunes. La evidencia acumulada sugiere que las exposiciones existentes a las cepas del resfriado común pueden proteger del desarrollo de síntomas graves en los infectados con SARS-CoV-2 [158]. Además de los CoV, las infecciones con otros virus respiratorios, las vacunas recientes contra la influenza y las vacunas infantiles con bacterias/virus vivos atenuados, como el bacilo de Calmette-Guérin (BCG) y la vacuna contra sarampión-paperas-rubeola (MMR) pueden ser parcialmente protectoras [159, 160, 161, 162]. Se desconocen los mecanismos biológicos exactos de la protección; sin embargo, un mecanismo epigenético que conduzca a la «inmunidad entrenada» de las células mieloides por las exposiciones previas a patógenos relacionados puede ser una causa plausible, de acuerdo con la información disponible en la literatura [163].

## Posible fisiopatología del «COVID persistente»

La persistencia o reaparición de los síntomas clínicos en forma de «COVID persistente» constituye un problema de salud importante en los pacientes dados de alta de COVID-19 [17, 18, 19]. Además de la persistencia o recurrencia de ciertos síntomas clínicos, se ha reportado un posible riesgo de infertilidad en varones, fatiga crónica y la aparición de diabetes en los supervivientes de COVID-19 [17, 19, 72, 164]. Además, se ha observado la formación de autoanticuerpos en muchos pacientes con COVID-19, lo que indica que los supervivientes pueden tener mayor riesgo de sufrir trastornos autoinmunes [149, 165]. La aparición de discapacidades [165, 166] y la probable reducción de la esperanza de vida, tal y como indican estudios recientes [167, 168], son preocupaciones importantes en los supervivientes. También se observan problemas de salud a largo plazo en la infección por otros BCoV, como el SARS-CoV-1 y el MERS-CoV [169]; sin embargo, aún no se conocen bien las razones. Aunque en la actualidad no se comprende claramente su fisiopatología, se han planteado diversas especulaciones con base en los hechos conocidos sobre las interacciones entre el virus y el huésped en el COVID-19. En general, la enfermedad crónica y la posterior escarificación y disfunción de los órganos afectados pueden ocasionar problemas de salud a largo plazo [167]. Además, la infección crónica por el SARS-CoV-2 puede inducir cambios epigenéticos en órganos vitales, reprogramando así sus funciones, como se demostró recientemente en un modelo murino de COVID-19, lo que puede preparar el terreno para los problemas de salud a largo plazo [38]. La inhibición radical del crecimiento celular en las células infectadas por el virus en los órganos puede ser otro mecanismo molecular importante que conduzca a problemas de salud a largo plazo [37]. La inhibición del crecimiento celular puede afectar especialmente a los tejidos con alta tasa mitótica, como los órganos reproductores y endocrinos, el epitelio vascular y de las mucosas, y las regiones cerebrales neurogénicas; por tanto, los problemas de salud, incluidos los relacionados con el aprendizaje y la memoria, pueden surgir a largo plazo en los supervivientes del COVID-19.

## El rostro cambiante de la pandemia: Cambio en las interacciones huésped-virus con las nuevas variantes del SARS-CoV-2

**Fig. 7:**
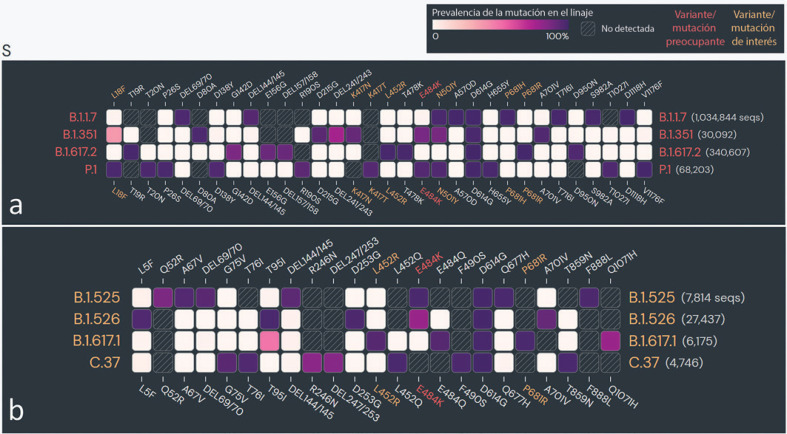
Mutaciones en la secuencia de codificación de la proteína spike en las variantes emergentes del coronavirus tipo 2 del síndrome respiratorio agudo severo (SARS-CoV-2) en el mundo. (**a**) Variantes preocupantes (VOC). (**b**) Variantes de interés (VOI). − El análisis incluyó mutaciones con prevalencia superior a 75% en al menos un linaje reconocido por la Organización Mundial de la Salud (OMS) como variante preocupante o de interés en todo el mundo. Fuente de datos: www.outbreak.info, consultado el 28/08/2021.

La amplia propagación de la primera oleada de COVID-19 más allá de sus límites geográficos habría creado una barrera inmunológica en la población infectada contra la cepa de tipo salvaje (WT) del SARS-CoV-2 (nCoV-2019), lo cual, se esperaba, limitaría las oleadas recurrentes. Paradójicamente, se produjeron nuevas oleadas masivas de COVID-19 impulsadas por variantes emergentes de SARS-CoV-2 en los años 2020–2021 en todo el mundo [170, 171, 172, 173]. Las variantes emergentes del SARS-CoV-2, que parecen tener mayor transmisibilidad y virulencia y son capaces de escapar a la inmunidad natural y adquirida (por vacunas y anticuerpos monoclonales utilizados terapéuticamente) contra las cepas WT [172, 173, 174, 175] (Tabla 2), han frustrado la esperanza de un final más rápido para la pandemia. Los recientes modelos in situ y en animales, así como los estudios clínicos, han confirmado que las variantes tienen un periodo de incubación más corto, una mayor carga viral y una excreción viral prolongada [176, 177, 178, 179, 180, 181]. Las variantes pueden causar mayores daños en el tejido del huésped infectado; sin embargo, aún se desconoce si ha habido cambios en el tipo de tejido que afectan o en la patogénesis específica en los órganos [172]. La OMS ha identificado cuatro variantes preocupantes (VOC) y cuatro variantes de interés (VOI) en el mundo [173]. Las variantes del SARS-CoV-2 se caracterizan por mutaciones clave específicas de cada linaje en las regiones de la proteína spike, y se considera que contribuyen a aumentar la transmisibilidad o la virulencia y el escape inmunitario a los anticuerpos naturales y adquiridos por la vacuna [3, 172, 176, 177] (Figura 7). Muchas de las mutaciones de la proteína spike son comunes a todas las variantes, lo que indica su evolución convergente y su ventaja selectiva para la aptitud epidemiológica [2] (Figura 7). Las mutaciones específicas del linaje también están presentes en las regiones no-spike de las variantes [182]; sin embargo, todavía se sabe poco sobre su importancia epidemiológica.

## Mecanismos plausibles para una mayor transmisibilidad/virulencia y capacidad de escape inmunológico en las variantes del virus

Se ha reportado un aumento en la transmisibilidad o la virulencia en casi todas las VOC y múltiples VOI [174] (Tabla 2). Ciertas regiones de la proteína spike contienen el sitio de unión con el receptor de entrada a la célula huésped y con los anticuerpos naturales y adquiridos del huésped. Se cree que algunas mutaciones clave en la proteína spike (Tabla 2), principalmente en el dominio de unión al receptor (RBD), inducen cambios conformacionales que dan lugar a una mayor unión al receptor de entrada a la célula huésped, ACE2 [2, 3]. Además, ciertas mutaciones han creado nuevos sitios de contacto, enlaces electrostáticos más fuertes o nuevos enlaces de hidrógeno entre el RBD y el ACE2 del huésped [2] (revisado en [176, 177]].

Es interesante que las mutaciones en la secuencia de la proteína spike de ciertas variantes (linaje B.1.1.7 y B.1.617) que se producen en la posición del aminoácido 681 (P-H/R), que cae en el FCS, mejoran la escisión proteolítica de la proteína spike, reforzando la fusión de la membrana viral con la membrana de la célula huésped (revisado en [1]). Se especula que una mejor fusión entre el virus y la célula huésped da lugar a una mayor formación de sincitios [1]. Se cree que la formación de sincicios, la fusión de la célula huésped infectada con otras células, facilita la propagación viral, lo que confiere mayor transmisibilidad a las variantes [183, 184, 185]. En particular, la formación de sincitios ha sido una característica distintiva del SARS-CoV-2 con respecto al SARS-CoV-1 (revisado en [1]).

Además del aumento en la transmisibilidad y la virulencia, estudios recientes han demostrado que la mayoría de las variantes, principalmente las VOC, han adquirido cierto nivel de resistencia contra la inmunidad natural y adquirida (por vacunas y anticuerpos monoclonales utilizados terapéuticamente) [174] (Tabla 2). Se han reportado frecuentes reinfecciones y escape de la inmunidad vacunal con las variantes [172, 186, 187]. Los mecanismos exactos de la adquisición de la capacidad de escape inmunitario en las variantes no se conocen bien. Sin embargo, según la literatura emergente, los mecanismos de escape inmunológico más probables son (i) la inclusión o eliminación de residuos de aminoácidos en los epítopos inmunogénicos (para los anticuerpos naturales y adquiridos), lo que conlleva cambios conformacionales en la interfaz de unión [2, 3, 4, 176, 177]; (ii) la remodelación del potencial de superficie electrostático en la interfaz de unión antígeno-anticuerpo [2, 3, 4, 176, 177]; y (iii) la adquisición de sitios de glicosilación adicionales, que protegen el sitio de unión con los anticuerpos neutralizantes [188]. Se necesitan más estudios para desentrañar los mecanismos que utilizan las variantes para escapar del sistema inmunitario.

## Observaciones finales

Se han llevado a cabo extensas investigaciones en todo el mundo para desentrañar los diversos mecanismos implicados en la patogénesis y las respuestas inmunitarias del huésped para el COVID-19. Estos estudios se han centrado principalmente en la proteómica y la genómica virales y en los factores dependientes del huésped. Se han realizado amplios estudios experimentales con modelos de COVID-19 basados en cultivos celulares y en animales. Además, se ha deducido una adaptación específica para el ser humano de los mecanismos de interacción entre el virus y el huésped a partir de los datos de laboratorio de pacientes con COVID-19 y de estudios experimentales con organoides de tejidos humanos. A la luz de los amplios descubrimientos realizados hasta ahora, tenemos un conocimiento amplio de las interacciones entre el virus y el huésped, el tropismo tisular y la patogénesis específica en los órganos, la participación de los sistemas fisiológicos y la respuesta inmunitaria humana contra la infección por el SARS-CoV-2. La expresión generalizada de los factores de entrada del SARS-CoV-2 en los tejidos humanos, la desregulación del SRAA y una respuesta inmunitaria innata hiperactiva acompañada de una inmunidad adaptativa retardada o suprimida parecen ser los factores clave de las manifestaciones sistémicas y la mala evolución clínica de los pacientes. La inclusión de FCS en la secuencia de la proteína spike puede ser una razón para el aumento de la virulencia del SARS-CoV-2. Además, la atenuación de la respuesta temprana al IFN y la subsiguiente tormenta de citocinas, así como una respuesta inmunitaria adaptativa suprimida/retrasada, marcada por una intensa linfocitopenia y una síntesis subóptima de inmunoglobulinas, son las características inmunológicas más destacadas, que parecen impulsar la gravedad de la enfermedad. Los factores genéticos preexistentes pueden estar detrás de la mayor vulnerabilidad de ciertos individuos a contraer la infección y de la agudización de la enfermedad; sin embargo, aún se sabe poco sobre esta cuestión. El microbioma intestinal también puede desempeñar un papel importante en la evolución de la enfermedad, tal y como indica la literatura emergente. Quedan múltiples lagunas en el conocimiento sobre varios aspectos de la enfermedad, que deben abordarse en futuros estudios. Además, la persistencia o recurrencia de los síntomas en forma de «COVID persistente» es una preocupación sanitaria seria, que debe investigarse a fondo. Y lo que es más importante, las variantes emergentes del SARS-CoV-2 que impulsan las oleadas recurrentes de COVID-19, causadas por el aumento de la transmisibilidad/virulencia y la capacidad de escape inmunitario, requieren una investigación más profunda para abordar con precisión su(s) mecanismo(s).

## Conflictos de interés

Los autores declaran que la investigación se llevó a cabo en ausencia de cualquier relación comercial o financiera que pudiera interpretarse como posible conflicto de interés.

## Información sobre licencias

Ashutosh Kumar, Ravi K. Narayan, Pranav Prasoon, Chiman Kumari, Gurjot Kaur, Santosh Kumar, Maheswari Kulandhasamy, Kishore Sesham, Vikas Pareek, Muneeb A. Faiq, Sada N. Pandey, Himanshu N. Singh, Kamla Kant, Prakash S. Shekhawat, Khursheed Raza, Sujeet Kumar: COVID-19 Mechanisms in the Human Body − What We Know So Far. Front Immunol. 2021 Nov 1;12:693938. ^©^ 2021 Los Autores (traducción; contribución de los autores, nota del editorial, agradecimientos abreviada), protegido por CC BY 4.0 (https://creativecommons.org/licenses/by/4.0/deed.es).

## Figures and Tables

**Table 1:** Diversidad sistémica de las manifestaciones clínicas en la enfermedad por coronavirus 2019 (COVID-19)

Sistema	Síntoma	Estudio
General	Fiebre	[6, 7]
	Cefalea	
	Fatiga	

Respiratorio	Tos seca	[6, 7]
	Dificultad respiratoria	
	Congestión nasal	
	Secreción nasal	
	Dolor de garganta	

SNC y órganos sensoriales	Psicosis aguda	[8, 9, 10, 11]
	Pérdida del sentido del olfato	
	Pérdida del sentido del gusto	
	Pérdida del habla	
	Vértigo	
	Deterioro de la conciencia	
	Accidente cerebrovascular	
	Ataxia	
	Convulsión	
	Deterioro de la visión
	Congestión ocular	
	Pérdida de audición, otalgia, vértigo, acúfenos	

Cardiaco	Dolor/presión torácica aguda	[12]
	Arritmia	
	Insuficiencia cardiaca	

Digestivo	Náusea y vómito	[6, 7, 13]
	Anorexia	
	Diarrea	
	Dolor abdominal	

Renal	Orina turbia con micción frecuente	[14]

Musculoesquelético	Mialgia	[6]

Piel, pelo y uñas	Erupción o cambio de color en los dedos de las manos o los pies Caída del cabello y calvicie Signo de lúnula roja en las uñas	[6, 15, 16]

**Table 2:** Variantes emergentes del coronavirus tipo 2 del síndrome respiratorio agudo severo (SARS-CoV-2) en todo el mundo y sus características clínico-epidemiológicas

Etiqueta OMS	Pango Linajes	Estado de la variante (OMS/CDC)	Clado GISAID	Clado Nextstrain	Mutaciones clave en Spike (frecuencia >75%)*	Primer reporte	Fecha de designación	Transmisión*	Letalidad*	Inmunoescape*
***Alfa***	B.1.17	Variante preocupante (VOC)^#^	GRY	20I (V1)	GRY 69del, 70del, 144del, E484K.S494P, N501 Y, A570D, D614G, P681H, T716I, S982A, D1118H K1191N	Reino Unido, Sep-2020	s18-Dic-2020	˜50% mayor transmisión comparada con B.1	Posible aumento de la gravedad con base en las tasas de hospitalización y de mortalidad	• Sin impacto en la susceptibilidad a tratamientos con anticuerpos monoclonales • Impacto mínimo en la neutralización por suero de convalecientes y vacunados

***Beta***	B.1.351 B.1.351.2 B.1.351.3	VOC#	GH/501Y.V2	20 H (V2)	69del, 70del, 144del, E484K, S494P, N501Y, A570D, D614G, P681H, T716I, S982A, D1118H, K1191N	Sudafrica, May-2020	18-Dic-2020	˜50% mayor transmisión	Más letal	• Reducción significativa en susceptibilidad a la combinación de anticuerpos monoclonales bamlanivimab y etesevimab • Reducción en la neutralización por suero de convalecientes y vacunados

***Gamma***	P.1 P.1.1 P.1.2 P.1.4 P.1.6 P.1.7	VOC^#^	GR/501Y.V3	20J (V3)	L18F, T20N, P26S, D138Y, R190S, K417T, E484K, N501Y, D614G, H655Y, T1027I	Brasil, Nov-2020	11-Ene-2021	Aún no determinada	Más letal	• Reducción significativa en susceptibilidad a la combinación de anticuerpos monoclonales bamlanivimab y etesevimab • Reducción en la neutralización por suero de convalecientes y vacunados

***Delta***	B.1.617.2 AY.1 AY.2 AY.3 AY.3.1	VOC^#^	G/478K.V1	21A	T19R,(G142D), 156del, 157del, R158G, L452R, T478K.D614G, P681R, D950N	India, Oct-2020	VOI:4-Abr-2021 VOC: 11-May-2021	˜50–60% mayor transmisión comparada con B.1.1.7	Resultados preliminares sugieren que el riesgo de hospitalización en 14 días es 2.61 veces mayor que el de B.1.1.7	• Reducción potencial de neutralización por algunos tratamientos con anticuerpos monoclonales • Reducción potencial de la neutralización por suero de pacientes vacunados

***Lambda***	C.37	Variante de interés (VOI)^#^	GR/452Q.V1	21G	D614G, L452Q, F490S, T859N, T76I, G75V, del247/253	Perú, Dic-2020	14-Jun-2021	Aún no determinada	Aún no determinada	• Aún no determinado

***Mu***	B.1.621, B.1.621.1	VOI^#^	GH	21H	Spike: T95I, Y144S, Y145N, R346K, E484K, N501 Y, D614G, P681H, D950N	Colombia, Ene-2021	Sep-2021	31-Aug-2021	Aún no determinada	• Aún no determinado

***Eta***	B.1.525	Variante bajo monitoreo (VOB)*	G/484K.V3	21D	A67V,69del, 70del, 144del, E484K.D614G, Q677H, F888L	Múltiples países, Dic-2020	17-Mar-2021	Aún no determinada	Aún no determinada	• Reducción potencial de neutralización por algunos tratamientos con anticuerpos monoclonales • Reducción potencial en la neutralización por suero de convalecientes y vacunados

***Iota***	B.1.526	Variante bajo monitoreo (VOB)*	GH/253G.V1	21F	L5F, T95I, D253G, S477N, E484K.D614G, A701V	Estados Unidos de América, Nov-2020	24-Mar-2021	Aún no determinada	Aún no determinada	• Reducción en susceptibilidad a la combinación de anticuerpos monoclonales bamlanivimab y etesevimab • Reducción en la neutralización por suero de convalecientes y vacunados

***Kappa***	B.1.617.1	Variante bajo monitoreo (VOB)*	G/452R.V3	21B	T95I.G142D, E154K, L452R, E484Q, D614G, P681R, Q1071H	India, Oct-2020	4-Abr-2021	Más transmisible	Mayor letalidad en un modelo animal. En humanos, aún no determinada	• Reducción potencial de neutralización por algunos tratamientos con anticuerpos monoclonales • Reducción potencial en la neutralización por suero de convalecientes y vacunados

# Basado en las últimas actualizaciones de la OMS, Ginebra [173].

* Basado en las últimas actualizaciones de los Centros para el Control y la Prevención de Enfermedades (CDC), Estados Unidos [174].
